# Emergence of acute quadriparesis in a young patient following myocardial infarction. A case report

**DOI:** 10.1002/ccr3.9366

**Published:** 2024-08-27

**Authors:** Sajjad Daneshyar, Mojtaba Khazaei, Mozhgan Nazifi

**Affiliations:** ^1^ Department of Neurology, School of Medicine Hamadan University of Medical Sciences Hamadan Iran

**Keywords:** arthritis rheumatoid, myocardial infarction, risk factors, stroke

## Abstract

Cerebrovascular events are closely related to cardiac events. Chronic inflammatory diseases, such as rheumatoid arthritis (RA), increase the risk of cardiovascular and cerebrovascular stroke. The link between RA, myocardial infarction, and resulting neurological issues highlights the case's complexity, stressing the need for a comprehensive, interdisciplinary medical approach.

## 1 INTRODUCTION

Myocardial infarction (MI) remains the primary cause of mortality among both men and women in the United States, with stroke ranking as the third leading cause of death and the primary contributor to disability.^1^ Acute coronary syndrome and cerebrovascular accidents (CVA) constitute medical emergencies that necessitate simultaneous diagnosis and treatment.^2^ Among the myriad factors contributing to MI, individuals with rheumatoid arthritis (RA) face an elevated risk of cardiovascular disease (CVD). Recent studies consistently highlight an increased risk of MI in individuals with RA compared to the general population.^3^ In this context, we present a case involving a patient with RA who initially exhibited early‐onset fatigue and nonspecific chest pain, leading to an acute MI. During hospitalization, the patient developed neurological symptoms, subsequently diagnosed as a cerebral stroke following the MI. This case underscores the significance of promptly addressing heart attacks to protect the brain and identifying neurological impairments through unusual symptoms for effective patient management.

## 2 CASE HISTORY/EXAMINATION

We introduce a 40‐year‐old female patient with a history of RA who visited the hospital emergency department following a cold, presenting with nonspecific symptoms of weakness and malaise. In the patient's drug history, the only medication noted was the use of corticosteroids, considering the history of RA. Physical examination revealed tachycardia (rate:128 beats/min, regular rhythm) and hypotension (blood pressure 75/50 mmHg).

## 3 METHODS

**FIGURE 1:**
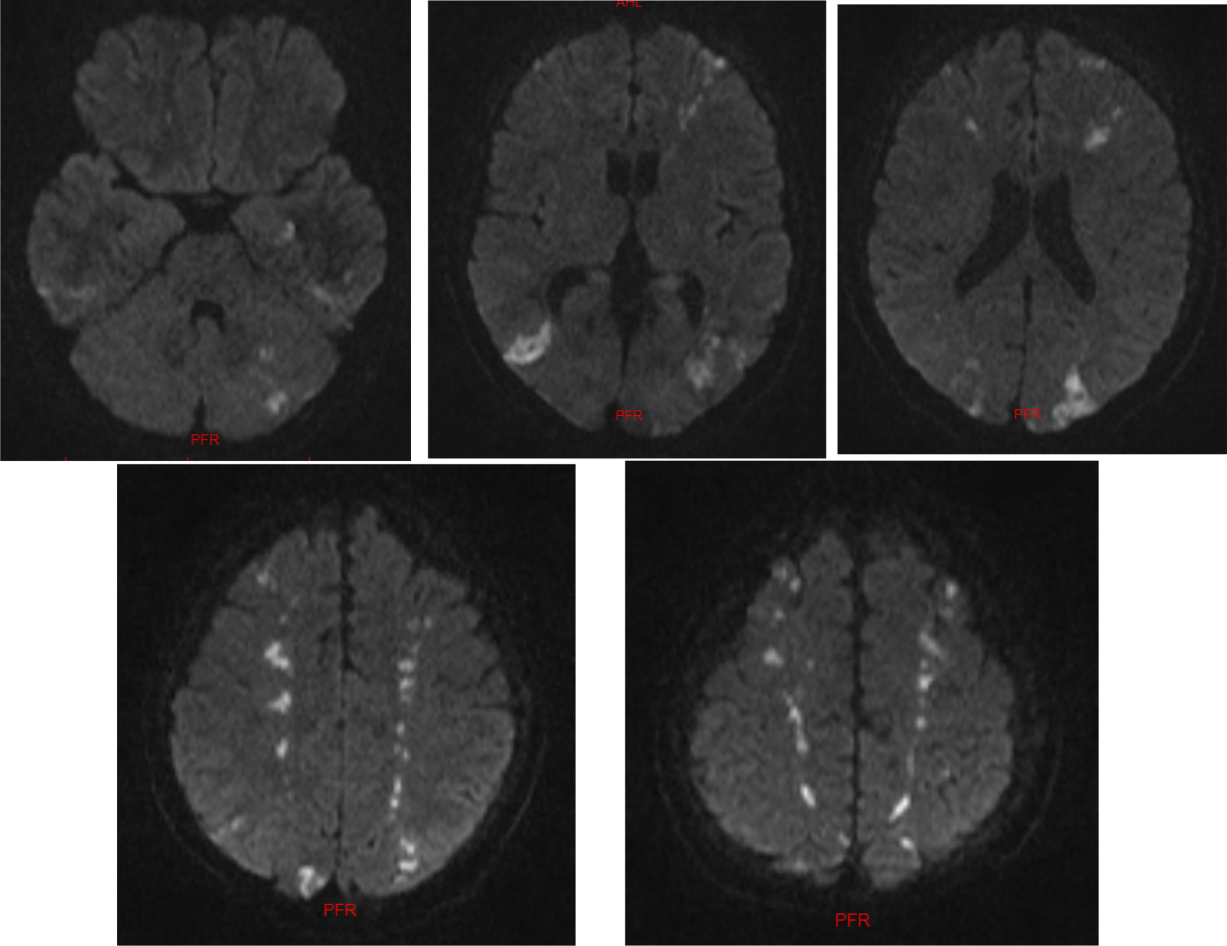
Axial section of brain MRI utilizing the DWI sequence, illustrating an acute watershed infarct involving both territories of the anterior and posterior cerebral vascular supplies.

Initial measures, including basic tests and an ECG, were conducted upon admission. During these investigations, sinus tachycardia and evidence of ST elevation were observed in leads V2‐4 and leads I and AVL on the patient's ECG, along with minimal reciprocal ST depression in leads III and AVF. The patient's cardiac troponin level was also measured at 172 ng/L. Liver function and other blood chemistries were normal. Diagnosed with an acute MI, the patient was admitted to the cardiac care unit, and according to the instructions from the cardiology service, the patient underwent appropriate medical management with proper doses of antiplatelets and statins, along with intravenous morphine. Additionally, they were considered a candidate for PCI (percutaneous coronary intervention) for reperfusion. In the early minutes of hospitalization, the patient developed neurological symptoms, manifesting as weakness in all four limbs, progressively worsening. Neurological consultation was promptly requested for the patient. Initially, the patient underwent a detailed neurological examination. In this examination, cranial nerves were intact. In bilateral plantar reflex examinations, an upward response was observed. Proximal motor strength in the lower limbs was three‐fifths, and in the upper limbs was two‐fifths. Given the upper motor neuron symptoms, The patient underwent emergency echocardiography, as well as brain and cervical CT scans. The patient's echocardiography reported an ejection fraction of 45% without LV thrombosis. For the cervical spine CT scan, considering the patient's history of RA, an assessment of the integrity of the C1 and C2 vertebrae was requested, which revealed no abnormalities. The brain CT scan revealed indistinct hypodensities in the parietal and occipital regions, suggestive of a stroke. Therefore, alongside continued treatment with antiplatelet and anticoagulant medications, as well as addressing the patient's hypotension through intravenous hydration, the patient underwent a brain MRI. (Figure 1).

In the brain MRI, as depicted in Figure 1, evidence of watershed cerebral infarction was observed in most territories within the anterior and posterior cerebral vascular supply, including the ACA‐MCA and MCA‐PCA territories. This pattern of involvement indicates a decrease in blood supply to the brain. It appears that in this patient, following a MI, systemic blood pressure has dropped, affecting both anterior and posterior cerebral circulation systems, leading to the occurrence of watershed infarction. Additionally, a brain CT angiography was conducted to evaluate intracranial vessels, revealing no significant stenosis in the intracranial arteries. The patient underwent medical treatment as well as physiotherapy and occupational therapy during the hospitalization. The strength in all four limbs showed improvement. Further examinations for the patient included bilateral transcranial Doppler ultrasonography, with no pathological findings reported. After completing the course of treatment and neurological assessments, the patient was transferred to the cardiology department for additional cardiac investigations.

## 4 CONCLUSIONS AND RESULTS

To prevent the occurrence of watershed cerebral infarctions, it is advisable to avoid sudden drops in blood pressure as much as possible. Endovascular interventions are recommended in the presence of any symptomatic stenosis in extracranial vessels. Additionally, if any acute neurological symptoms arise from cardiac causes, a neurological examination is warranted.

## 5 DISCUSSION

Watershed infarctions are acute ischemic strokes that predominantly occur in regions of the brain known as border zones, between the two blood supply territories.^4^ Two hypotheses exist to determine the mechanism of watershed infarction, with one attributing it to decreased blood flow to brain tissue (hypoperfusion). Reports indicate instances of syncope preceding watershed infarctions, supporting the hypoperfusion hypothesis. Another theory considered for the mechanism of watershed infarction is the micro embolism infarct theory. It suggests that unstable atherosclerotic plaque in arterial walls leads to micro emboli formation, resulting in multiple infarcts in brain tissue. Imaging and pathological studies suggest that embolic infarctions predominantly affect distal cortical vessels.^5^ Accurate diagnosis of watershed infarction, like other infarctions, involves using MRI. Brain CT angiography (CTA) examines major intracranial vessels and small arteries. CTA enables a more in‐depth investigation of the causes of watershed infarction for therapeutic and preventive measures, including assessing risk factors for CVAs.^6^ RA, mentioned in our patient's medical history due to its inflammatory nature, predisposes individuals to a higher incidence of vascular diseases compared to the general population, such as MIs and vasculitis.^3^ A reduction in cerebral blood flow, for any reason, can disrupt the autoregulation system, leading to cerebral metabolic disturbances. Causes for this decrease in cerebral blood flow that can result in watershed infarction include syncope or other cardiac issues such as arrhythmias or MIs. In our studied patient, a drop in blood pressure and decreased cerebral blood flow following a MI set the stage for watershed infarction in both anterior and posterior cerebral vascular systems. Most patients with watershed infarctions have favorable outcomes. However, cases, where infarction occurs post‐cardiac surgery, have a higher probability of bilateral watershed infarction and often have poorer outcomes. It is reported that 8 out of 10 cases of mortality in watershed infarctions occur post‐cardiac surgery.^7^ Non‐surgical patients usually have unilateral infarctions, often accompanied by gradual arterial stenosis leading to collateral formation. In our study patient, despite experiencing both MI and bilateral watershed cerebral infarctions, there was a favorable prognosis, with improved muscle strength and other neurological symptoms during hospitalization.

## AUTHOR CONTRIBUTIONS

**Sajjad Daneshyar:** Data curation; writing – original draft. **Mojtaba Khazaei:** Project administration. **Mozhgan Nazifi:** Writing – original draft.

## FUNDING INFORMATION

None.

## CONFLICT OF INTEREST STATEMENT

The authors declare no conflict of interest.

## ETHICAL APPROVAL

Patient's informed written consent was obtained.

## CONSENT

Written informed consent was obtained from the patient to publish this report in accordance with the journal's patient consent policy.

## Data Availability

The data used to support this study are included within the article. Further inquires can be directed to the corresponding author.
